# Contractibility of a persistence map preimage

**DOI:** 10.1007/s41468-020-00059-7

**Published:** 2020-08-28

**Authors:** Jacek Cyranka, Konstantin Mischaikow, Charles Weibel

**Affiliations:** 1 Department of Mathematics, Rutgers, The State University of New Jersey, 110 Frelinghusen Rd., Piscataway, NJ 08854-8019 USA; 2 Institute of Informatics, University of Warsaw, Banacha 2, 02-097 Warsaw, Poland

**Keywords:** Topological data analysis, Persistent homology, Dynamical systems, Fixed point theorem, 37C25, 55N31, 06B35, 55-08, 06F30

## Abstract

This work is motivated by the following question in data-driven study of dynamical systems: given a dynamical system that is observed via time series of persistence diagrams that encode topological features of snapshots of solutions, what conclusions can be drawn about solutions of the original dynamical system? We address this challenge in the context of an *N* dimensional system of ordinary differential equation defined in ${\mathbb {R}}^N$. To each point in ${\mathbb {R}}^N$ (e.g. an initial condition) we associate a persistence diagram. The main result of this paper is that under this association the preimage of every persistence diagram is contractible. As an application we provide conditions under which multiple time series of persistence diagrams can be used to conclude the existence of a fixed point of the differential equation that generates the time series.

## Introduction

*Topological data analysis* (TDA), especially in the form of persistent homology, is rapidly developing into a widely used tool for the analysis of high dimensional data associated with nonlinear structures (Edelsbrunner and Harer 2010; Zomorodian and Carlsson 2005; Oudot 2015). That topological tools can play a role in this subject should not be unexpected, given the central role of nonlinear functional analysis in the study of geometry, analysis, and differential equations, for example. What is perhaps surprising is that, to the best of our knowledge, there have been no systematic attempts to rigorously analyze the dynamics of differential equations using persistent homology.

Persistent homology is often used as a means of data reduction. A typical example takes the form of a complicated scalar function defined over a fixed domain, where the geometry of the sub-(super)-level sets is encoded via homology. Of particular interest to us are settings in which the scalar function arises as a solution to a partial differential equation (PDE); we are interested in tracking the evolution of the function, but experimental data only provides information on the level of digital images of the process. Furthermore, capturing the dynamics of a PDE often requires a long time series of rather large digital images. Thus, rather than storing the full images, one can hope to work with a time series of persistence diagrams. Our aim is to draw conclusions about the dynamics of the original PDE from the time series of the persistence diagrams. This is an extremely ambitious goal and far beyond our capabilities at the moment. A much simpler question is the following: if there is an attracting region in the space of persistence diagrams, under what conditions can we conclude that there is a fixed point for the PDE?

This paper represents a first step towards answering the simpler question. Theorem 4.3 shows that given an ordinary differential equation (ODE) with a global compact attractor ${{\mathcal {A}}}\subset {\mathbb {R}}^N$ and a neighborhood in the space of persistence diagrams that is mapped into itself under the dynamics, then there exists a fixed point for the ODE. In applications one could consider the ODE as arising from a finite difference approximation of the PDE.

The challenge is that to obtain results one must understand the topology of $data_P$, the space of data having a fixed persistence diagram *P*, a topic for which there are only limited results. That the structure of $data_P$ is complicated follows directly from the fact that persistent homology can provide tremendous data reduction, but in a highly nonlinear fashion. With this in mind, the primary goal of this paper is to show that for a reasonable class of problems the space $data_P$ is a finite set of contractible, simplicial sets. The importance of this result is that it opens the possibility of applying standard algebraic topological tools, e.g., Lefschetz fixed point theorem, Conley index, to dynamics that is observed through the lens of persistent homology.

To state our goal precisely requires the introduction of notation. Throughout this paper ${{\mathcal {S}}_N}$ denotes the 1-dimensional simplicial complex composed out of *N* vertices [*i*] ($i=1,\ldots ,N$) and $N-1$ edges $[i,i+1]$ ($i=1,\ldots ,N-1$). It is a simplicial decomposition of closed bounded interval in ${\mathbb {R}}$.

We study filtrations of ${{\mathcal {S}}_N}$ defined as follows.

### Definition 1.1

Let $z=(z_1,\ldots ,z_N)\in {\mathbb {R}}^N$. Define $f:{\mathbb {R}}^N\times {{\mathcal {S}}_N}\rightarrow {\mathbb {R}}$ by

$$\begin{aligned} f(z,\sigma ) := {\left\{ \begin{array}{ll} z_j &{}\text {if } \sigma =[j],\\ \max \left\{ {z_j, z_{j+1}}\right\} &{}\text {if } \sigma =[j,j+1]. \end{array}\right. } \end{aligned}$$

For $r\in {\mathbb {R}}$, we set ${{\mathcal {S}}_N}(z,r) := \left\{ {\sigma \in {{\mathcal {S}}_N}: f(z,\sigma )\le r}\right\} .$

### Definition 1.2

Given $z=(z_1,\ldots ,z_N)\in {\mathbb {R}}^N$, we can reorder the coordinates of *z* such that

$$\begin{aligned} z_{j_1} \le z_{j_2} \le \cdots \le z_{j_N}. \end{aligned}$$

The *sublevel-set filtration of* ${{\mathcal {S}}_N}$ *at* *z*,^1^ which we write as ${{\mathcal {S}}_N^{{{\mathsf {F}}}}}(z)$, is given by

$$\begin{aligned} {{\mathcal {S}}_N}(z,z_{j_1}) \subseteq {{\mathcal {S}}_N}(z,z_{j_2}) \subseteq \cdots \subseteq {{\mathcal {S}}_N}(z,z_{j_N}). \end{aligned}$$

Because ${{\mathcal {S}}_N^{{{\mathsf {F}}}}}(z)$ is a finite filtration of simplicial complexes, completely determined by *z*, we can use classical results from (Edelsbrunner and Harer 2010; Zomorodian and Carlsson 2005) to compute the persistence diagram of ${{\mathcal {S}}_N^{{{\mathsf {F}}}}}(z)$. We treat this as a map

$$\begin{aligned} \mathsf {Dgm}:{\mathbb {R}}^N\rightarrow \mathsf {Per}, \end{aligned}$$

where $\mathsf {Per}$ denotes the space of all persistence diagrams. Thus the space $data_P$ of all $z\in {\mathbb {R}}^N$ having persistence diagram *P* is just $\mathsf {Dgm}^{-1}(P)$. We remark that there are a variety of topologies that can be put on $\mathsf {Per}$ such that $\mathsf {Dgm}$ becomes a continuous map (Chazal et al. 2016; Cohen-Steiner et al. 2007).

Since ${{\mathcal {S}}_N}$ is one-dimensional and contractible, we are only concerned with the persistent homology $H_0$, i.e., the persistence diagrams associated with connected components. Therefore for the rest of the paper we restrict our study to consist of the family $\mathsf {Per}$ of persistence diagrams of level zero.

Here is the main result of this paper.

### Theorem 1.3

For every persistence diagram *P*, the space $data_P\subset {\mathbb {R}}^N$ is composed of a finite number of mutually disjoint components. Each component is contractible, and is homeomorphic to a finite union of convex, potentially unbounded polytopes.

The proof of Theorem 1.3 is not particularly difficult, but it is technical. We first describe the connected components of $data_P$; see Lemma 2.4. In Sect. 2.2, we introduce the poset $\mathsf {Str}$ of cellular strings, which are be used to decompose each component as a finite union of convex polytopes in Sect. 2.3. In Sect. 3, we show that the realization of $\mathsf {Str}$ is contractible.

**Fig. 1:**
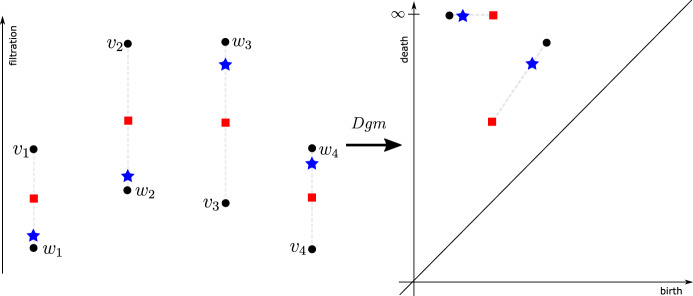
Non-convexity of the preimage $data_P$ under the persistence map $\mathsf {Dgm}$. In the left figure the two vectors, $v=(v_1,\ldots ,v_4)$ and $w=(w_1,\ldots ,w_4)$, lie in the preimage of the persistence diagram *P*, composed out of two black points visible on the right figure. Applying $\mathsf {Dgm}$ to convex linear combinations of *v* and *w* results in a path in the persistence plane illustrated on the right (the convex path is marked in grey, and two sample vectors on the path are marked using red squares and blue stars) (color figure online)

To emphasize that Theorem 1.3 is not a trivial result, we use Fig. 1 to demonstrate that $data_P$ is not a convex subet of ${\mathbb {R}}^N$. In particular, consider the vectors $v=(v_1,\ldots , v_4)$ and $w=(w_1,\ldots , w_4)$ on the left of Fig. 1. It is left to the reader to check that $\mathsf {Dgm}(v)=\mathsf {Dgm}(w)$ and that this persistence diagram is given by the pair of black dots (see right of Fig. 1). Note that the vectors in ${\mathbb {R}}^4$, indicated (on the left) in blue stars and red squares, lie on a straight line from *v* to *w*. However, the persistence diagrams indicated (on the right) in blue and red clearly differ from $\mathsf {Dgm}(v)$. Thus, the red and blue vectors do not lie in $data_{\mathsf {Dgm}(v)}$.

In Sect. 4 we apply Theorem 1.3 to prove the existence of fixed points with given persistence diagrams for a dissipative ordinary differential equation.

## Invariants for a fixed persistence diagram

Fix a persistence diagram *P*. To describe the structure of the space $data_P$, we introduce two levels of invariants: the critical value sequences, representing the connected components of $data_P$, and (for each of these), a partially ordered set $\mathsf {Str}$ indexing a polytope decomposition of the component.

### Components

Fix a persistence diagram *P*. To describe the (finitely many) connected components of $data_P$, it is useful to introduce notation that records the order in which the relevant local maxima and minima occur.

We say that $z=(z_1,\ldots ,z_N)\in {\mathbb {R}}^N$ is a *typical point* if its coordinates are distinct. If *z* is a typical point and $1<n<N$, we say that $z_n$ is a *local minimum* (of *z*) if $z_{n-1}>z_n <z_{n+1}$, and a *local maximum* if $z_{n-1}<z_n >z_{n+1}$; it is a *local extremum* if it is a local minimum or maximum. We say that $z_1$ and $z_N$ are *boundary extrema*; $z_1$ is a local minimum (resp., maximum) if $z_1<z_2$ (resp., $z_1>z_2$).

#### Definition 2.1

The *critical value sequence* of a typical point $z=(z_1,\ldots ,z_N)$ is

$$\begin{aligned} {{\,\mathrm{cv}\,}}(z) = \left( z_{n_1},\ldots , z_{n_K}\right) \in {\mathbb {R}}^K, \end{aligned}$$

where the $z_{n_k}$ are the local extrema of *z*, excluding boundary extrema that are local maxima, and $n_1< n_2< \cdots < n_K$.

#### Example 2.2

Let $z=(1.5,-0.9,1.1,2.1,1.4)\in {\mathbb {R}}^5$. The local minimum is $z_2$ and the local maximum is $z_4$. The boundary extrema are $z_1$ and $z_5$. Since $z_1$ is also a local maximum we do not include it in the critical value sequence. Thus $h={{\,\mathrm{cv}\,}}(z) = (-0.9,2.1,1.4)$.

The following notion emphasizes the structure of the critical value sequences.

#### Definition 2.3

A 010 *critical value sequence* of (odd) length *K* is a vector $\text {cv}=(z_1,\dots ,z_K)\in {\mathbb {R}}^K$ with the property that

$$\begin{aligned} z_{n_1}< z_{n_2}> z_{n_3}< \cdots < z_{n_{K-1}} >z_{n_K}. \end{aligned}$$

A 101 *critical value sequence* is defined similarly, with the inequalities reversed.

Since we are using sublevel set filtrations to compute the persistence diagram we focus on 010 critical value sequences.

Lemma 2.4 below shows that the local extrema of *z* are determined up to order by its persistence diagram, and hence that there are only finitely many critical value sequences for any fixed persistence diagram.

Recall that a persistence diagram is a finite collection of *persistence points* $\left\{ {p_i = (p_i^b, p_i^d)}\right\}$, where $p_i^b$ and $p_i^d$ denote birth and death values, respectively. Since ${{\mathcal {S}}_N}$ is connected, the persistence diagram of a typical point *z* has a unique persistence point $p_i=(p_i^b,p_i^d)$ such that $p_i^b=\min _{n=1,\ldots , N}z_n$ and $p_i^d = \infty$; without loss of generality, we may relabel $p_i$ as $p_1$.

#### Lemma 2.4

Let $z\in {\mathbb {R}}^N$ be a typical point with persistence diagram

$$\begin{aligned} \left\{ {p_m = (p_m^b, p_m^d) \mid m=1,\ldots ,M}\right\} . \end{aligned}$$

Then, *z* has $K=2M-1$ local extrema; the local minima of *z* are precisely $\left\{ {p_m^b}\right\} _{m=1}^M$ and the interior local maxima of *z* are precisely $\left\{ {p_m^d}\right\} _{m=2}^M$.

We leave the proof of Lemma 2.4 to the reader, remarking that it still holds when $z\in {\mathbb {R}}^N$ is not a typical point, except that the persistence diagram may be a multiset (there may be multiple copies of a single persistence point).

Given a point *z* with persistence diagram *P*, let *C*(*z*) denote the component of $data_P$ containing *z*.

The following lemma shows that $data_P$ is the disjoint union of the finitely many disjoint components *C*(*z*), indexed by the critical value sequences. The proof follows from the observation that the order of the local extrema cannot be changed while preserving the persistence diagram.

#### Lemma 2.5

If *z* and $z'$ are typical points in ${\mathbb {R}}^N$ then $C(z)=C(z')$ if and only if ${{\,\mathrm{cv}\,}}(z) = {{\,\mathrm{cv}\,}}(z')$.

Moreover, *C*(*z*) is the closure of the set of typical points in *C*(*z*).

This proves the first assertion in Theorem 1.3.

#### Remark 2.6

The components *C*(*z*) group vectors into equivalence classes that can be characterized using the notion of *chiral merge tree* as defined in Curry (2018). Corollary 5.5 of Curry (2018) shows that the number of chiral merge trees realizing diagram *P* is equal to $2^{N-1}\prod _{j=2}^N{\mu _B(I_j)}$, where *B* is the barcode realization of *P*, i.e. set of intervals $I_j= [b_j,d_j]$ having the birth and death values of the *j*-th persistence point as its endpoints, and $\mu _B(I_j)$ is the number of intervals in *B* that contain $I_j$.

### Cellular strings

In this section, we define the poset $\mathsf {Str}(N,M)$ of *cellular strings* associated to *M* points arising from a vector in ${\mathbb {R}}^N$. Thus we fix *N* and *M*, where $N\ge 2M-1$.

Consider a string of symbols $s=s_1\cdots s_N$ of length *N*, where each symbol $s_n$ is either 0, 1, or *X* (we refer to 0 and 1 as *bits*). Any such string can be represented as $s=\gamma _1\cdots \gamma _J$ where each block $\gamma _j$ is a substring made up of a single symbol (that is, $\gamma _j$ is $0\cdots 0$, $1\cdots 1$, or $X\cdots X$), and consecutive blocks have different symbols. We refer to $s=\gamma _1\cdots \gamma _J$ as the *canonical representation* of *s*.

#### Definition 2.7

Fix $M<N$. A 010 *cellular string*^2^ is a symbol string *s* of length *N* such that, for the canonical representation $s=\gamma _1\cdots \gamma _J$:

(i) the symbols that make up $\gamma _j$ and $\gamma _{j+1}$ are different;
(ii) $\gamma _1$ and $\gamma _J$ consist of the symbols 0 or *X*;
(iii) if $\gamma _j$ consists of the symbol *X*, then the symbol of $\gamma _{j-1}$ is different from the symbol of $\gamma _{j+1}$;
(iv) there are exactly *M* values of *j* for which $\gamma _j$ consists of the symbol 0.

The set $\mathsf {Str}(N,M)$ of cellular strings is a poset, where $s'< s$ if the string *s* is obtained from $s'$ by replacing some of the bits 0 and 1 in $s'$ by *X*.

The *dimension* of a cellular string *s*, $\dim (s)$, is the number of symbols *X* in *s*. It follows from (iv) that *M* of the blocks $\gamma _j$ have the form $0\cdots 0$, and $M-1$ have the form $1\cdots 1$. Thus, $K=2M-1$ of the blocks are bitstrings. If these bitstrings are $\gamma _{j_1}, \ldots , \gamma _{j_K}$, then the symbol for $\gamma _{j_k}$ is 0 if *k* is odd and 1 if *k* is even. Since each block has at least one symbol, it follows that any cellular string has dimension at most $L=N-K$.

We write $\mathsf {Str}^{(r)}(N,M)$ for the sub-poset of all cellular strings whose first $r-1$ symbols are *X*. Note that $\mathsf {Str}(N,M) = \mathsf {Str}^{(1)}(N,M)$ and $\mathsf {Str}^{(L+1)}(N,M)=\{X\cdots X010\cdots 10\}$.

#### Proposition 2.8

An element of Str(*N*, *M*) is maximal if and only if it is an *L*-dimensional cellular string, where $L = (N-K)$.

#### Proof

Let $s=\gamma _1\dots \gamma _J\in \mathsf {Str}(N,M)$. By definition, $\dim (s)\le L$. Conversely, suppose that the symbol *X* appears in *s* has less than *L* times. Then some bitstring $\gamma _j$ has length $\ge 2$. Let $s'$ be the cellular string obtained by replacing the first symbol of $\gamma _j$ by *X*. Then $s<s'$, so *s* is not maximal. $\square$

**Fig. 2:**
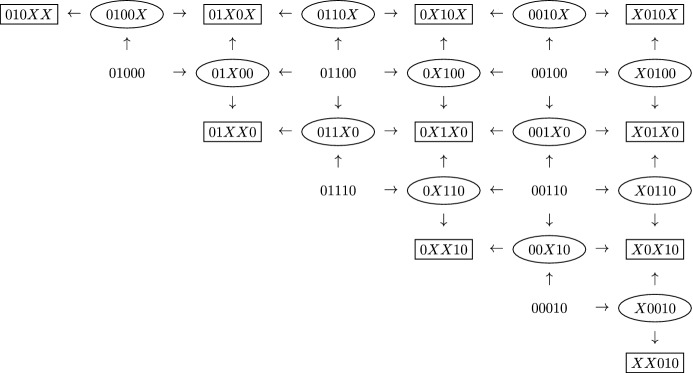
The string poset $\mathsf {Str}$ for $M=2$ and $N=5$. Two-dimensional, one-dimensional, and zero-dimensional strings are surrounded by rectangles, ellipses, and nothing, respectively. The arrows indicate the partial order. The rightmost column is the sub-poset $\mathsf {Str}^{(2)}$

Since both *N* and *M* are fixed in our analysis, we simplify the notation and write $\mathsf {Str}$ for $\mathsf {Str}(N,M)$. Figure 2 illustrates the poset $\mathsf {Str}$ when $M=2$, $K=3$ and $N=5$; the right column is $\mathsf {Str}^{(2)}$.

#### Lemma 2.9

Every string $s'$ is the greatest lower bound of the set of *L*-dimensional strings *s* with $s'<s$.

It follows that $\mathsf {Str}$ has the least upper bound property: if two strings have a lower bound, they have a greatest lower bound.

#### Proof

We proceed by downward induction on the dimension *d* of $s'$, the case $d=L$ being clear. Consider the canonical representation, $s'= \gamma _1\cdots \gamma _J$. If $d<L$, then some bitstring $\gamma _j$ has length $\ge 2$. Consider the strings $s_1=\gamma _1\cdots \gamma _{j-1}X{\bar{\gamma }}\gamma _{j+1}\cdots \gamma _J$ and $s_2=\gamma _1\cdots \gamma _{j-1}{\bar{\gamma }} X\gamma _{j+1}\cdots \gamma _J$ where ${\bar{\gamma }}$ is a bitstring consisting of the same symbol as $\gamma _j$ but of length one less than $\gamma _j$. Since this is the form of any cellular string *s* satisfying $s'<s$ and $\dim s = \dim s' +1$, the result follows. $\square$

Let *s* be an *L*-dimensional cellular string. Successively replacing an *X* adjacent to a bit (0 or 1) by that bit yields a chain of strings $s=s_L>s_{L-1}>\cdots>s_1>s_0$. It follows that every maximal chain in the poset has length *L*.

#### Example 2.10

Consider a string $s(n)=\sigma _10X\cdots X1\sigma _2$ with a block of *n* consecutive *X*’s (where $\sigma _1$ and $\sigma _2$ are fixed substrings). Let $\mathsf {Str}/s(n)$ denote the sub-poset of $\mathsf {Str}$ consisting of all strings $s'\le s(n)$ which begin in $\sigma _10$ and end in $1\sigma _2$. Then $\mathsf {Str}/s(n)$ is isomorphic to the poset $I_n$ of integer intervals [*i*, *j*] with $1\le i\le j\le n+1$. (The string corresponding to [*i*, *j*] is

$$\begin{aligned} \sigma _10\cdots 0X\cdots X1\cdots 1\sigma _2; \end{aligned}$$

it has *i* 0’s and the first 1 is in the $(j+1)^{st}$ spot.)

If *s* is a cellular string with *k* blocks of successive *X*’s (of lengths $n_1,\ldots ,n_k$), the sub-poset $\mathsf {Str}/s$ of strings $s'<s$ in $\mathsf {Str}$ is isomorphic to the product of posets $\mathsf {Str}/s(n_1),\ldots ,\mathsf {Str}/s(n_k)$, i.e., to the poset

$$\begin{aligned} I_{n_1}\times \cdots \times I_{n_k}. \end{aligned}$$

### The polytopes

We now turn to identifying the polytopes of Theorem 1.3. Fix a 010 critical value sequence ${{\,\mathrm{cv}\,}}= \left( z_{n_1},\ldots ,z_{n_K}\right)$ as in Definition 2.3. To each *d*-dimensional cellular string *s* we assign a *d*-dimensional polytope *T*(*s*) in ${\mathbb {R}}^N$; *T*(*s*) will be a product of simplices.

Let $s = \gamma _1\gamma _2\cdots \gamma _J$ be the canonical representation of a string *s*, as in Definition 2.7. Let $n_j$ denote the length of the substring $\gamma _j$, so $N=\sum n_j$.

- If $\gamma _j$ is either $0\cdots 0$ or $1\cdots 1$, and $\gamma _j$ is the $k^{th}$ block from the left involving 0 or 1, we set
  $$\begin{aligned} T(\gamma _j) = \left\{ {z_k}\right\} ^{n_k} = (z_k,\ldots ,z_k). \end{aligned}$$
- If $\gamma _1$ is a block $X\cdots X$, then
  $$\begin{aligned} T(\gamma _1) = \left\{ { (x_1,\ldots ,x_{n_j})\in {\mathbb {R}}^{n_j}: \infty \ge x_1\ge \cdots \ge x_{n_1} \ge z_1 }\right\} . \end{aligned}$$
- If $\gamma _J$ is a block $X\cdots X$, then
  $$\begin{aligned} T(\gamma _J) = \left\{ { (x_1,\ldots ,x_{n_j})\in {\mathbb {R}}^{n_j}: z_k\le x_1\le \cdots \le x_{n_1} \le \infty }\right\} . \end{aligned}$$
- If $\gamma _j$ is a block $X\cdots X$ (for $1< j < J$), and $\gamma _{j-1}$ is the $k^{th}$ block from the left involving 0 or 1, then
  $$\begin{aligned} T(\gamma _j)= & {} \left\{ { (x_1,\ldots ,x_{n_j})\in {\mathbb {R}}^{n_j}}\right\} \quad \text {where}\quad \\&{\left\{ \begin{array}{ll} z_k \le x_1 \le \cdots \le x_{n_j}\le z_{k+1} &{} \text {if } k \hbox { is odd}; \\ z_k \ge x_1 \ge \cdots \ge x_{n_j}\ge z_{k+1} &{}\text {if } k \hbox { is even}. \end{array}\right. } \end{aligned}$$
- We define $T(s)\subset {\mathbb {R}}^N$ to be the concatenation:
  $$\begin{aligned} T(s) = T(\gamma _1\gamma _2\cdots \gamma _J) = \prod _{j=1}^J T(\gamma _j). \end{aligned}$$

Let *P* be a persistence diagram and $z\in dgm^{-1}(P)$. The component *C*(*z*) of $data_P$ is the union of the T(s), where $s\in \mathsf {Str}$ and *T*(*s*) is defined using the critical value sequence ${{\,\mathrm{cv}\,}}(z)$. This is clear from Definition 2.1.

Since the critical value sequence is always assumed to be fixed, we will suppress it in the notation.

#### Example 2.11

Consider the case $K=3$ and $N=5$. If $s=01XX0$, then $(\gamma _1,\ldots ,\gamma _4) = (0,1,XX,0)$. So, $(n_1,n_2,n_3,n_4)=(1,1,2,1)$ and hence

$$\begin{aligned} T(01XX0)= & {} \left\{ {z_1}\right\} \times \left\{ {z_2}\right\} \times \left\{ {(x_1,x_2) : z_2 \ge x_1 \ge x_2 \ge z_3}\right\} \times \left\{ {z_3}\right\} \\\cong & {} \Delta ^0\times \Delta ^0\times \Delta ^2\times \Delta ^0. \end{aligned}$$

If $s=X01X0$, then $(\gamma _1,\ldots ,\gamma _4,\gamma _5) = (x,0,1,x,0)$. So, $(n_1,n_2,n_3,n_4,n_5)=(1,1,1,1,1)$ and hence

$$\begin{aligned} T(X01X0)= & {} [z_1,\infty )\times \left\{ {z_1}\right\} \times \left\{ {z_2}\right\} \times [z_3,z_2]\times \left\{ {z_3}\right\} \cong [0,\infty )\\&\times \Delta ^0\times \Delta ^0\times \Delta ^1\times \Delta ^0 \end{aligned}$$

Similarly, $$T(X0100) = [z_1,\infty )\times \left\{ {z_1}\right\} \times \left\{ {z_2}\right\} \times \left\{ {z_3}\right\} \times \left\{ {z_3}\right\} \cong [0,\infty )\times \Delta ^0\times \Delta ^0\times \Delta ^0\times \Delta ^0$$.

Observe that $X0100 <X01X0$ and $T(X0100)\subset T(X01X0)$.

Let $\mathbf {Poly}$ denote the poset of polytopes in ${\mathbb {R}}^N$ under inclusion. By definition, *T* maps strings in $\mathsf {Str}$ to polytopes in $\mathbf {Poly}$.

#### Lemma 2.12

*T* :  Str$\rightarrow \mathbf {Poly}$ is an injective poset morphism, and preserves greatest lower bounds.

#### Proof

Suppose that $s'<s$ and $1+\dim s' = \dim s$. If $s' = \gamma _1\cdots \gamma _J$ is the canonical form, then some $\gamma _j$ has the form $a\cdots a$ (where *a* is 0 or 1), and *s* has the form

$$\begin{aligned} s_1=\gamma _1\cdots {\bar{\gamma }}_jX \cdots \gamma _J \quad \text {or}\quad s_2=\gamma _1\cdots X{\bar{\gamma }}_j \gamma _J, \end{aligned}$$

where ${\bar{\gamma }}_j=a\cdots a$ has one fewer bit that $\gamma _j$. It is clear from the definition of *T* that $T(s_1)\ne T(s_2)$, and $T(s')$ is the intersection of $T(s_1)$ and $T(s_2)$, as desired. $\square$

### Geometric realization of posets

Let *C* be a poset (partially ordered set). For any $c\in C$, we write *C*/*c* for the sub-poset $\left\{ {c':c'\le c}\right\}$; *C* is the union of the *C*/*c*. If $c_1$ and $c_2$ have a greatest lower bound $c_{12}$, then $(C/c_1)\cap (C/c_2)=C/c_{12}$.

By definition, the geometric realization *BC* of any poset *C* is a simplicial complex whose *k*-dimensional simplices are indexed by the chains $c_0<c_1<\cdots c_k$ of length *k* in *C*. It is the union of the realizations *B*(*C*/*c*) of the sub-posets *C*/*c*; if $c_1$ and $c_2$ have a greatest lower bound $c_{12}$, then $B(C/c_1)$ and $B(C/c_2)$ intersect in $B(C/c_{12})$. See Weibel (2013, IV.3.1) for more details.

Here are some basic facts; see Weibel (2013, IV.3) for a discussion. A poset morphism $f:C\rightarrow C'$ determines a continuous map $BC\rightarrow BC'$, and a natural transformation $\eta :f\Rrightarrow f'$ between morphisms gives a homotopy $B\eta :BC\rightarrow BC'$ between *f* and $f'$. In addition, realization commutes with products: $B(C_1\times C_2)\cong (BC_1)\times (BC_2).$ Applying these considerations to the poset $\mathsf {Str}$, we see that its realization $B\mathsf {Str}$ is the union of the polytopes $B(\mathsf {Str}/s)$, and if $s_{12}$ is the greatest lower bound of $s_1$ and $s_2$ then $B(\mathsf {Str}/s_1)\cap B(\mathsf {Str}/s_2)$ is $B(\mathsf {Str}/s_{12})$.

Let *s* be a cellular string. We saw in Example 2.10 that the poset $\mathsf {Str}/s$ is isomorphic to the product $I_{n_1}\times \cdots \times I_{n_k}$ of the posets $I_{n_j}$ of integer intervals in $[1,n_j+1]$, corresponding to the blocks of $n_j$ succesive *X*’s in *s*. It is well known that $B(I_n)$ is homeomorphic to the *n*-simplex $\Delta ^n$. Thus

$$\begin{aligned} B(\mathsf {Str}/s) \cong \prod B(I_{n_j}) \cong \Delta ^{n_1} \times \cdots \times \Delta ^{n_k}. \end{aligned}$$

By construction, $T(s)=\prod T(\gamma _j)$ also has this form. Hence we have a natural homeomorphism

$$\begin{aligned} B(\mathsf {Str}/s) \cong \prod B(\mathsf {Str}/s(n_j)) \cong \prod B(I_{n_j}) \cong \prod T(\gamma _j) = T(s). \end{aligned}$$

#### Theorem 2.13

*B* Str is homeomorphic to *C*(*z*).

#### Proof

By construction, $C(z) = \bigcup T(s)$, and $B\mathsf {Str}= \bigcup B(\mathsf {Str}/s)$. It suffices to observe that for each $s_1,\ldots ,s_n$ the restriction of the $B\mathsf {Str}/s_i \cong T(s_i)$ induces a homeomorphism between the intersection of the $B(\mathsf {Str}/s_i)$ and the intersection $T(s_i)$. This holds because the two sides are identified with $B(\mathsf {Str}/s')$ and $T(s')$, where $s'$ is the greatest lower bound of the $s_i$. $\square$

## Contractibility

We now define a poset morphism $F_1:\mathsf {Str}\rightarrow \mathsf {Str}$, and modify it to define poset morphisms $F_\ell :\mathsf {Str}^{(\ell )}\rightarrow \mathsf {Str}^{(\ell )}$ for $\ell >1$.

### Definition 3.1

Let *s* be an *L*-dimensional cellular string. We define $F_1(s)$ to be the string obtained from *s* by transposing the first (i.e., leftmost) *X* with the bit immediately preceding it. If *X* is the initial symbol, we set $F_1(s)=s$.

If *s* is a lower-dimensional cellular string, we define $F_1(s)$ as follows. If *s* has an initial *X* with no 00 or 11 preceding it, we do as before: transpose *X* with the bit immediately preceding it, or do nothing if *X* is the initial symbol. If *s* begins with a block of $n+1$ zeroes, say $s=00\cdots 0\sigma _2$, we replace the initial 0 by *X*, so $F_1(s)=X0\cdots 0 \sigma _2$. Otherwise, the string must have the form $s'=\sigma _1abb\sigma _2$, where *a*, *b* are bits, $a\ne b$, $\sigma _1$ is an (alternating) bitstring not ending in *a*, and $\sigma _2$ is the remainder of the string. We set

$$\begin{aligned} F_1(s')=\sigma _1aab\sigma _2. \end{aligned}$$

The definition of $F_\ell :\mathsf {Str}^{(\ell )}\rightarrow \mathsf {Str}^{(\ell )}$ mimics that of $F_1$. Specifically, if $s=\beta \sigma$, where $\beta =X\cdots X$ is a block of length $\ell -1$ then $F_{\ell }(s) = \beta F_1(\sigma )$.

### Example 3.2

In Fig. 2, the map $F_1$ sends strings surrounded by rectangles (resp., ellipses) from one column to strings surrounded by rectangles (resp., ellipses) in the second column to the right, while leaving the last column fixed. Thus $F_1(01100)= 00100$ and $F_1(00100)=X0100$.

Since $\mathsf {Str}^{(2)}$ is the rightmost column, the map $F_2$ acts on this column, mapping strings surrounded by rectangles (resp., ellipses) to those two rows down. Thus $F_2(XX010)=XX010$, $F_2(X0010)=XX010$, and $F_2(XX010)=XX010$.

### Lemma 3.3

$F_1:$ Str$\rightarrow \mathsf {Str}$ is a poset morphism, and is the identity on the sub-poset $\mathsf {Str}^{(2)}$.

Furthermore, $$F_1^K(\mathsf {Str}) = \mathsf {Str}^{(2)}$$.

### Proof

We proceed by downward induction on $d=\dim (s)$ to show that if $s'<s$ then $F_1(s')\le F_1(s)$. If $s'$ contains an *x* with no 00 or 11 preceeding it, the same is true for *s* and the inequality is evident.

Next, suppose that $s'=\sigma _1abb\cdots b\sigma _2$, where $\sigma _1a$ is an alternating bitstring. If $s = \sigma _1abb\cdots b\sigma '_2$ for some $\sigma _2\le \sigma '_2$ then

$$\begin{aligned} F(s') =\sigma _1aab\cdots b\sigma _2 \quad < \quad F(s)=\sigma _1aab\cdots b\sigma '_2. \end{aligned}$$

For $s_1=\sigma _1aXb\cdots$ and $s_2=\sigma _1ab\cdots bX\sigma _2$, we also have $F(s')<F(s_1)$ and $F(s')<F(s_2)$. Otherwise, either $s_1 < s$ or $s_2 < s$; in these cases, $F_1(s_1)\le F_1(s)$ or $F_1(s_2)\le F_1(s)$, by induction, and hence $F(s')<F(s)$.

Finally, if $s'=00\cdots 0\sigma$ then either $s_1=X0\cdots 0\sigma \le s$ or else $s_2=00\cdots 0X\sigma \le s$. By induction, $F_1(s_1)\le F_1(s)$ or $F_1(s_2)\le F_1(s)$, so it suffices to observe that $F_1(s')\le F_1(s_1), F_1(s_2).$ $\square$

### Remark 3.4

The proof of Lemma 3.3 also shows that each $F_\ell$ is a poset morphism.

We can filter the poset $\mathsf {Str}$ by sub-posets $Fil_i$, where $Fil_0=\mathsf {Str}^{(2)}$, $Fil_K=\mathsf {Str}$ and $Fil_i$ is the full poset on the set of strings *s* with $F_1^i(s)\subset \mathsf {Str}^{(2)}$. In Fig. 2, for example, $Fil_1$ (resp., $Fil_2$) is the rightmost 3 columns (resp., 5 columns). Since $F_1$ maps $Fil_i$ to $Fil_{i-1}$, the geometric realization of $BF_1$ restricts to a continuous map from $BFil_i$ to $BFil_{i-1}$. We will prove:

### Proposition 3.5

The inclusions $BFil_{i-1}\subseteq BFil_i$ are homotopy equivalences. Hence *B* Str$^{(2)}\subseteq B$ Str is a homotopy equivalence.

### Proof

For $i>0$, we define poset morphisms $F_{1,i}:Fil_i\rightarrow Fil_{i-1}\subseteq Fil_i$ to be the identity on $Fil_{i-1}$ and $F_1$ otherwise. The geometric realization of $F_{1,i}$ is a continuous map $BFil_i\rightarrow BFil_{i-1}\subseteq BFil_i$ which is the identity on $BFil_{i-1}$.

We will prove that, on geometric realization, $BF_{1,i}$ is homotopic to the identity on $BFil_i$.

We define a poset morphism $h:Fil_i\rightarrow Fil_i$ as follows. If $s\in Fil_{i-1}$ then $h(s)=s$; if $s\not \in Fil_{i-1}$, define *h*(*s*) to be the greatest lower bound of *s* and $F_1(s)$. Thus *Bh* is a continuous map from $BFil_i$ to itself. For $s\in Fil_i$, the inequalities $s \ge h(s) \le F_{1,i}(s)$ yield natural transformations $\text {id}_i {\Leftarrow } h \Rrightarrow F_1$. and hence homotopies between the maps $\text {id}_i$ (the identity map on $BFil_i$), *Bh* and $BF_{1,i}$. $\square$

### Corollary 3.6

Each *B* Str$^{(\ell +1)}\subset B$ Str$^{(\ell )}$ is a homotopy equivalence. In particular, the inclusion of the point *B* Str$^{(L+1)}$ in *B* Str is a homotopy equivalence, i.e., $B\mathsf {Str}$ is contractible.

### Remark 3.7

We can describe the map $T(s)\rightarrow T(F_1(s))$ induced by $F_1$. For example, suppose that $s=\sigma _1\gamma _{j-1}\gamma _j\sigma _2$, where $\sigma _1=\gamma _1\cdots \gamma _{j-1}$ is an alternating bitstring of length $\ge 2$ and $\gamma _j$ is a block $X\cdots X$. Then $T(\gamma _{j-1}) = \{z_{j-1}\}$ and $T(\gamma _j)\subset {\mathbb {R}}^{n_j}$ is defined by inequalities, either $z_{j-1}\le x_1\cdots$ or $z_{j-1}\ge x_1\cdots$, depending on the parity of *j*. The map $F_{1}$ sends $T(\gamma _{j-1})\times T(\gamma _j)$ to the subset

$$\begin{aligned} T(X)\times \{z_{j-1}\}\times T(\gamma '), \end{aligned}$$

where *T*(*X*) is defined by $z_{j-2}\le x_1\le z_{j-1}$ and $T(\gamma ')$ is defined by the equations $z_{j-1}\le x_2\cdots$ or $z_{j-2}\ge x_1\cdots$. In effect, the map sends $x_1$ to $z_{j-1}.$

## Existence of fixed points for flows

As an application of Theorem 1.3, we establish the existence of a fixed point solution of a ordinary differential equation whose trajectories are being observed in the space of persistence diagrams. To be more precise consider a differential equation ${\dot{z}} = f(z)$, $z\in {\mathbb {R}}^N$, with the property that it possesses a compact global attractor ${{\mathcal {A}}}$ (Raugel 2002). Given an initial condition $z(0)={\bar{z}}\in {\mathbb {R}}^N$, we write $z(t)=\varphi (t,{\bar{z}})$, $t\in [0,\infty )$ for the solution in forward time. The important consequence of the existence of a compact global attractor is that there exists $R>0$ such that for any initial condition ${\bar{z}}$ there exists $t_{{\bar{z}}}>0$ such that $\Vert \varphi (t,{\bar{z}}) \Vert < R$ for all $t\ge t_{{\bar{z}}}$. We say that *R* is a *bound* for ${{\mathcal {A}}}$. Observing the persistence diagrams along a trajectory results in a curve $\mathsf {Dgm}(\varphi (t,{\bar{z}}))\in \mathsf {Per}$. In what follows we do not assume that we have knowledge of the nonlinearity of *f*, or of the actual trajectories $\varphi (t,z)$; we are only given the curves $\mathsf {Dgm}(\varphi (t,{\bar{z}}))$ of persistence diagrams.

Even if the persistence diagram is constant, we cannot conclude that the underlying differential equation has a fixed point. As an example, consider a differential equation in ${\mathbb {R}}^3$ with a periodic solution in which the first coordinate $z_1=0$ is constant, and $(z_2,z_3)$ oscillates with the property that $1\le z_2 \le z_3$. The associated curve in $\mathsf {Per}$ consists of the constant persistence diagram $P=\left\{ {(0,\infty )}\right\}$.

However, Theorem 4.3 provides a scenario under which the observation of sufficiently many trajectories suggests the existence of a fixed point for the unknown ordinary differential equation that generates the dynamics. More general theorems are possible and, as will be discussed in a later paper, these techniques can be lifted to the setting of partial differential equations defined on bounded intervals. The purpose of this example is to emphasize the importance of Theorem 1.3 from the perspective of data analysis. Thus, we focus on a much more modest result. We will show that if a particular type of neighborhood in $\mathsf {Per}$ is positively invariant under the dynamics, i.e. if $\mathsf {Dgm}(z)$ is in the neighborhood implies that $\mathsf {Dgm}(\varphi (t,z))$ is in the neighborhood for all $t>0$, then there exists a fixed point for the differential equation that generates the dynamics. To state and obtain such a result requires the introduction of additional notation.

### Definition 4.1

We shall say that a persistence diagram

$$\begin{aligned} P = \left\{ {p_m=(p_m^b,p_m^d): m = 1,\ldots , M}\right\} \end{aligned}$$

is *sparse* if each persistence point is unique, i.e. $p_m \ne p_n$ for all $m\ne n$.

Given a sparse persistence diagram we can choose $\mu >0$ such that $\Vert p_m-p_n\Vert _\infty \ge 4\mu$ for all $m\ne n$ and $|p_m^d - p_m^b| \ge 4\mu$ for all *m*.

### Example 4.2

**Fig. 3:**
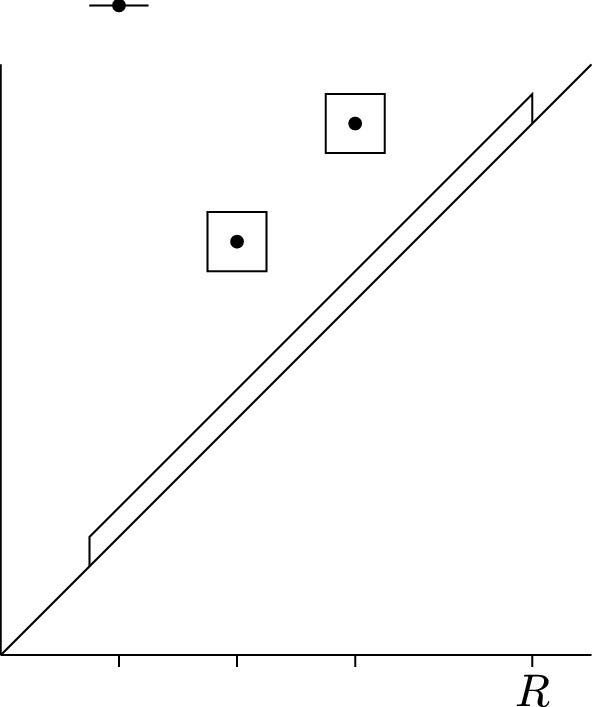
A sparse persistence diagram *Q* with persistence points $\left\{ {(1,\infty ),(2,3.5),(3,4.5)}\right\}$. The boxes indicate the set ${{\mathsf {N}}}_Q$ for $\mu = 0.25$

A sparse persistence diagram *Q* is shown in Fig. 3. We can choose $\mu = 0.25$. A possible critical value sequence associated to *Q* is ${{\,\mathrm{cv}\,}}(z)=(3,4.5,1,3.5,2)$.

We use $\mu$ to define subsets of ${\mathbb {R}}^N$ and $\mathsf {Per}$. We begin by constructing a subset of ${\mathbb {R}}^N$ using the set of cellular strings $\mathsf {Str}(N,M)$. Choose a point ${\hat{z}}$ with persistence diagram *P*. This gives rise to a fixed critical value sequence ${{\,\mathrm{cv}\,}}({\hat{z}})$ and the associated component $C({\hat{z}})\subset {\mathbb {R}}^N$ of $data_P$ is given by

$$\begin{aligned} C({\hat{z}}) = \bigcup _{s\in \mathsf {Str}(N,M)} T(s). \end{aligned}$$

By Theorem 1.3, $C({\hat{z}})$ is a contractible union of polytopes.

Let $B_\mu (C({\hat{z}}))\subset {\mathbb {R}}^N$ be the set of points that lie within a distance $\mu$ of $C({\hat{z}})$ using the $\sup$-norm. The bound on the choice of $\mu$ guarantees that if $s',s''\in \mathsf {Str}(N,M)$ are of maximal dimension and there does not exist $s\in \mathsf {Str}(N,M)$ such that $s<s'$ and $s<s''$, then $B_\mu (T(s'))$ and $B_\mu (T(s''))$ are disjoint. Therefore $B_\mu (C({\hat{z}}))$ is contractible.

We now turn to the subset of $\mathsf {Per}$. For each $m=1,\ldots , M$ set

$$\begin{aligned} {{\mathsf {P}}}_m := \left\{ {p=(p^b,p^d) : \Vert p-p_m\Vert _1 \le \mu }\right\} \end{aligned}$$

and

$$\begin{aligned} {{\mathsf {D}}}:= \left\{ {p=(p^b,p^d) : p^b \in [p_1^b-\mu , R]\ \text {and}\ 0\le p^d-p^b\le \mu }\right\} \end{aligned}$$

for some $R > \sup \{p^b_m\}+\mu$. See Fig. 3. Define ${{\mathsf {N}}}_P \subset \mathsf {Per}$ to be the set of persistence diagrams generated by elements of ${\mathbb {R}}^N$ with the property that for each $m=1,\ldots , M$ there exists a unique persistence point in ${{\mathsf {P}}}_m$ and any other persistence points lie in ${{\mathsf {D}}}$.

These constructions allow us to prove the following theorem concerning the existence of fixed points of the unknown, underlying dynamical system $\varphi$.

### Theorem 4.3

Consider a dynamical system generated by an ordinary differential equation that has a global compact attractor ${{\mathcal {A}}}$ with a bound *R*, and whose trajectories are represented by $\varphi (t,z)$. Let *P* be a sparse persistence diagram and let ${{\mathsf {N}}}_P\subset \mathsf {Per}$ be defined as above. Assume that if Dgm$(z)\in {{\mathsf {N}}}_P$, then Dgm$(\varphi (t,z))\in {{\mathsf {N}}}_P$ for all $t\ge 0$.

Then, for each component of Dgm$^{-1}({{\mathsf {N}}}_P)\subset {\mathbb {R}}^N$ there exists a vector ${\hat{z}}$ such that Dgm$({\hat{z}})\in {{\mathsf {N}}}_P$ and $\varphi (t,{\hat{z}})={\hat{z}}$ for all $t\in {\mathbb {R}}$, i.e. ${\hat{z}}$ is a fixed point for the dynamical system.

### Proof

We begin with the observation that if $z\in B_\mu (C({\hat{z}}))$ and there exists $t_1 >0$ such that $\varphi (t_1,z)\not \in B_\mu (C({\hat{z}}))$, then there exists $t_0 \in (0,t_1]$ such that $\mathsf {Dgm}(\varphi (t_0,z))\not \in {{\mathsf {N}}}_P$. This follows from the stability theorem of persistent homology using the bottleneck distance (Cohen-Steiner et al. 2007). This contradicts the hypothesis, therefore, that $B_\mu (C({\hat{z}}))$ is a contractible, positively invariant region under the dynamics. By McCord and Mischaikow (1996, Proposition 3.1) the Conley index of the maximal invariant set is that of a hyperbolic attracting fixed point. By McCord (1988, Corollary 5.8) (which utilizes the well known Lefschetz fixed point theorem), the maximal invariant set in $B_\mu (C({\hat{z}}))$ contains a fixed point. $\square$

## Conclusion and future work

Recall from Remark 2.6 that Curry (2018) provides a count of the contractible components of the preimage of a persistence map. However, to the best of our knowledge, this paper provides the first detailed analysis of the homotopy type of these of a components. Although we have presented the results in the context of sublevel set filtrations, the same arguments can be applied in the setting of superlevel set filtrations. The only significant change is that one needs to use 101 cellular strings; see Definitions 2.3 and 2.7.

Theorem 4.3, and the use of persistence diagrams to obtain results about the dynamics of an ODE, may appear somewhat artificial. However, consider a PDE, such as a reaction diffusion equation, defined on an interval. A finite spatial sampling of the solution at a time point gives rise to a vector. We can think of this vector as arising from two different proceedures: (i) numerical, e.g. the values of an ODE derived from a Galerkin approximation to the PDE, or (ii) experimental, e.g. a pixelated image of the solution. Theorem 4.3 is applicable in both cases, and one expects that for fine enough discretization or resolution that the results of Theorem 4.3 will be applicable to the PDE. The example involving images brings us much closer to current treatments of complex spatio-temporal dynamics (Kramar et al. 2016; Levanger et al. 2019). Hence, the natural next step in our research is to obtain an analogous result about existence of fixed points for one-dimensional PDEs whose trajectories are observed in the persistence space.

Finally, the obvious open question as a result of this paper is: given a *d*-dimensional simplicial complex ${\mathcal {S}}$ with a function *f*, similar in form to that of Definition 1.1, can one determine the homology of components of the pre-image of a persistence diagram?

## Footnotes

1 Analogous results can be obtain for superlevel set filtrations (see Sect. 5).

2 A 101 cellular string is defined similarly, interchanging 0 and 1.

## References

[CR1] Chazal, F., de Silva, V., Glisse, M., Oudot, S.: The Structure and Stability of Persistence Modules. Springer Briefs in Mathematics. Springer International Publishing AG Switzerland (2016)

[CR2] Cohen-Steiner D, Edelsbrunner H, Harer J (2007). Stability of persistence diagrams. Discret. Comput. Geom..

[CR3] Curry J (2018). The fiber of the persistence map for functions on the interval. J. Appl. Comput. Topol..

[CR4] Edelsbrunner H, Harer J (2010). Computational Topology.

[CR5] Kramar M, Levanger R, Tithof J, Suri B, Xu M, Paul M, Schatz MF, Mischaikow K (2016). Analysis of Kolmogorov flow and Rayleigh–Benard convection using persistent homology. Phys. D-Nonlinear Phenom..

[CR6] Levanger R, Xu M, Cyranka J, Schatz MF, Mischaikow K, Paul MR (2019). Correlations between the leading Lyapunov vector and pattern defects for chaotic Rayleigh–Benard convection. Chaos: Interdiscip. J. Nonlinear Sci..

[CR7] McCord, C.: Mappings and homological properties in the Conley index theory. Ergod. Theory Dynam. Syst. **8***(Charles Conley Memorial Issue), 175–198 (1988)

[CR8] McCord C, Mischaikow K (1996). On the global dynamics of attractors for scalar delay equations. J. Am. Math. Soc..

[CR9] Oudot, S.Y.: Persistence Theory: From Quiver Representations to Data Analysis. Mathematical Surveys and Monographs, vol. 209. American Mathematical Society, Providence (2015)

[CR10] Raugel G, Fiedler B (2002). Global attractors in partial differential equations. Handbook of dynamical systems.

[CR11] Weibel CA (2013). The $$K$$-book.

[CR12] Zomorodian A, Carlsson G (2005). Computing persistent homology. Discret. Comput. Geom..

